# Editorial: The Challenges and Opportunities of Introspection in Psychology: Theory and Method

**DOI:** 10.3389/fpsyg.2019.02196

**Published:** 2019-10-09

**Authors:** Ulrich W. Weger, Johannes Wagemann, Christian Tewes

**Affiliations:** ^1^Department of Psychology, Witten/Herdecke University, Witten, Germany; ^2^School of Psychology, University of Kent, Canterbury, United Kingdom; ^3^Institute for Waldorf Education, Inclusion and Intercultural Studies in Mannheim, Alanus University of Arts and Social Sciences, Alfter, Germany; ^4^Heidelberg University, Heidelberg, Germany

**Keywords:** consciousness, introspection, qualitative research, first-person science, methodology

Since the inception of psychology as an academic discipline, scholars have made extensive use of first-person (or introspective) enquiry: it is applied during theory-development and hypothesis-formulation; it permeates the data-collection stage; and it impacts on the process of data-interpretation and theory-development. In all these cases, the scientists' expertise, understanding, perspective, or expectations inform their scholarly practice. Sometimes this is the case in more explicit ways; sometimes, however, it can also occur in more implicit forms—and in turn it can color and even bias the research process.

This universally applied impromptu or naïve form of introspection can thus be a burden to the research process and we hence considered it important to raise this issue more broadly. We were not only seeking to draw attention to the widely practiced, often unreflected and hence problematic form of first-person enquiry; rather, we were also hoping to advance methods that could systematically develop and advance this practice so that it can become a form of enquiry that may complement other, third-person forms of research. This was the methodological aim of our Research Topic.

The other, theoretical aim was to explore more subtle facets of psychological phenomena that are difficult to access from a third-person point of view. Many such phenomena have first-person characteristics or are even of a primary first-person nature to begin with (such as the self; the qualia of consciousness; or the experiential aspect of illness) and it appears inadequate to try and explore these topics while bypassing what a first-person exploration can uncover. First- and third-person research should not be seen as competing approaches but as providing complementary access routes in a pluralistic research culture.

To bring together the available expertise and to explore current approaches on what is admittedly still a niche-theme—often even marred with notorious skepticism and apodictic rejection—we compiled the current Research Topic. From a competitive selection of contributions, six were successful in the review process and in what follows we provide a brief overview to orient the reader through these articles.

In his paper *Is there a problem in the laboratory?* Wendt voices reservations against the common practice of reducing problem-solving research to laboratory settings, as this does not give justice to the experiential facets of many real-life problem-solving situations. Wendt proposes a phenomenologically oriented understanding and definition of what a problem is and proposes a set of empirical strategies that a revised approach should consider. These include the use of less intrusive methods of enquiry; and the inclusion of “atmospheric” characteristics of the experimental setup, for instance via the consideration of artwork.

In his contribution *The Phenomenology of Habits: Integrating First-Person and Neuropsychological Studies of Memory*, Tewes explores the possibility to integrate first-person research on embodied memory capacities with third-person research methods as commonly used in neuropsychology. Starting with the concept of habits, he analyzes phenomenological and embodied work of habitual memory capacities in order to show that next to rigid and stereotypical habits there are also flexible and adaptive ones, which are open to attentional control and accessible from the first-person perspective. In the last part of the paper he outlines how the method of front-loaded phenomenology—developed by Shaun Gallagher—could contribute to exploring this type of habitual body memory also with neurophysiological methods.

In his article *The relevance of explanatory first-person approaches for understanding psychopathological phenomena. The Role of Phenomenology*, Schmidt draws on the Husserlian concept of genetic phenomenology to illuminate different psychopathological phenomena. He points to the importance of complementing biological/reductionist accounts of psychiatric conditions with phenomenological ones, as many experiential facets of psychiatric disorders have correlate symptoms that cannot be immediately traced back to the primary physiological deficiency. He points out how phenomenological research needs to be used to systematize psychopathological experiences rather than understand them via the detour of physiology.

In his article *Husserlian phenomenology as a kind of introspection*, Gutland introduces phenomenology as a form of introspection and gives an overview over Husserl's phenomenological approach. He reviews the challenges of introspective enquiry and points to safeguards that Husserl introduced to shield phenomenological introspection against these challenges. One is the systematic scrutiny and reduction of accounts to those aspects that are fully compatible with what one experiences. The other is the exploration of different (imaginary) instantiations of a phenomenon and the attempt to identify patterns of lawfulness that permeate these—an approach Husserl termed eidetic variations. Gutland discusses the implications of these aspects and also places Husserl's account in the wider philosophical context.

In his article *The confluence of perceiving and thinking in consciousness phenomenology*, Wagemann focuses on the phenomenon that similar dynamic patterns of separation and integration appear in the history of human consciousness as well as in elementary mental processes. Against this background—and substantiated with representative examples—he complements the standard approach to perception and thought as externally measurable processes with systematic first-person observations inspired by Steiner's and Witzenmann's Structure Phenomenology. In the context of voluntary perceptual reversals, this allows the author to trace back perceiving and thinking action to a diachronic structure of separating and integrating mental micro-gestures and to relate this to the historical development of psychology.

In their article *Pristine inner experience and descriptive experience sampling: Implications for psychology*, Lesley-Carr and Heavey define pristine inner experience as experiences that is spontaneous and not yet colored by interpretation. Such experiences are theoretically important as they allow for an unobtrusive assessment of a momentary psychological state. The authors introduce descriptive experience sampling as a method to enquire into these spontaneously available states and illuminate how this method can help uncover facets of psychological functioning that are difficult to access with other methods.

Taken together, the Research Topic has implications for basic research as well as applied (clinical) practice and will equip the reader with an overview over the challenges and opportunities of introspection—and how these can be addressed and/or made use of.

## Author Contributions

All authors listed have made a substantial, direct and intellectual contribution to the work, and approved it for publication.

### Conflict of Interest

The authors declare that the research was conducted in the absence of any commercial or financial relationships that could be construed as a potential conflict of interest.

